# Halved Dose of Antipsychotics Versus High-Dose Antipsychotic Therapy for Relapse in Patients with Schizophrenia Receiving High-Dose Antipsychotic Therapy: A Randomized Single-Blind Trial

**DOI:** 10.3390/ijms26094003

**Published:** 2025-04-23

**Authors:** Ryota Ataniya, Takeshi Koike, Atsuko Inamoto

**Affiliations:** 1 Showa University Northern Yokohama Hospital, 35-1, Chigasakichuou, Tsuzuki-Ku, Yokohama-Shi 224-0032, Japan; 2 Department of Psychiatry, Edogawa Hospital, 2702, Yamazaki, Noda-Shi 278-0022, Japan

**Keywords:** schizophrenia, high-dose antipsychotic therapy, relapse, dopamine D2 receptor, inverted U-shaped dose response

## Abstract

Both a shortage and an excess of dopamine (DA) in the prefrontal cortex and striatum result in their decreased functions, and the relationship between the DA levels and their functions exhibits an inverted-U shape. Increased DA transmission via dose reduction in the currently used antipsychotics may improve the activation of DA-related symptoms in schizophrenia; these include delusions and auditory hallucinations caused by increased DA release. In this case, reducing the dose of the antipsychotic may be a treatment option for relapse in patients with schizophrenia who are already on high doses of antipsychotics and find it difficult to further increase the dose. A total of 54 inpatients with schizophrenia receiving high-dose antipsychotic therapy were randomly assigned to either the halved-dose group or the high-dose group (symptomatic treatment). The study compared the time from relapse to improvement between the two groups. In the halved-dose group, the period until relapse improvement ranged from 1 to 3 weeks, while the high-dose group experienced improvement in 4 to 9 weeks, and a significant difference was observed between the two groups using Kaplan–Meier survival analysis (*p* < 0.001).

## 1. Introduction

The abrupt discontinuation of antipsychotics in patients with schizophrenia who have not achieved sufficient remission, even those receiving high-dose antipsychotic treatment, has demonstrated rapid improvement in psychiatric symptoms [1,2,3,4]. The relationship between dopamine (DA) stimulation and DA-related functions exhibits an inverted U-shaped curve. When the DA levels are either too low or too high, there is a reduction in the DA-related functions, such as reward-based learning and motivated behavior [5,6,7,8]. It is inferred that positive symptoms of schizophrenia, closely related to DA levels [9,10,11,12], are similarly reduced when DA stimulation is too low or too high. However, the abrupt discontinuation of antipsychotics may lead to rebound psychosis, withdrawal akathisia, withdrawal dyskinesia, and sleep disturbances, requiring the reinstatement of antipsychotic medication to improve symptoms [1,4]. Antipsychotic discontinuation (or medication non-adherence) is the largest factor contributing to relapse in schizophrenia treatment [13,14]. Opting to halve rather than discontinue antipsychotic doses appears to be an appropriate approach for effectively improving symptoms while minimizing the withdrawal effects. Laruelle et al. showed that increased DA release in the striatum is associated with the worsening of positive symptoms and relapse [15], and they characterized relapse as a hyperdopaminergic state, i.e., a situation where presynaptic dopamine function is augmented, indicating an increase in DA synthesis and DA stores. In human PET studies, stress-induced DA release in the striatum was higher in individuals with a risk of psychosis and in patients with schizophrenia compared with healthy controls [16,17,18], indicating that elevated DA synthesis in the striatum correlates with the worsening of psychotic symptoms and impaired cognitive function [19,20]

**Figure 1:**
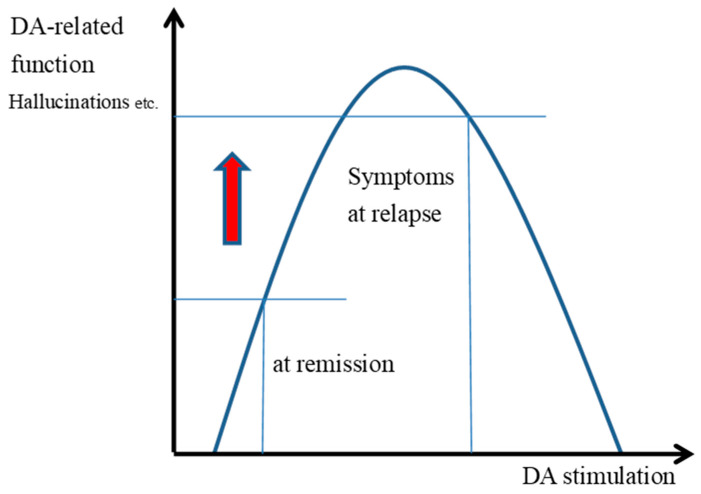
Changes in dopamine stimulation and function at remission and relapse in patients with schizophrenia.

In patients with schizophrenia during periods of remission, hallucinations and delusions occur less frequently, and impulsive action is reduced (Figure 1).

When relapse occurs, increased DA stimulation causes an increase in erroneous and inappropriate impulsive actions (Figure 1: the red arrow in the diagram).

**Figure 2:**
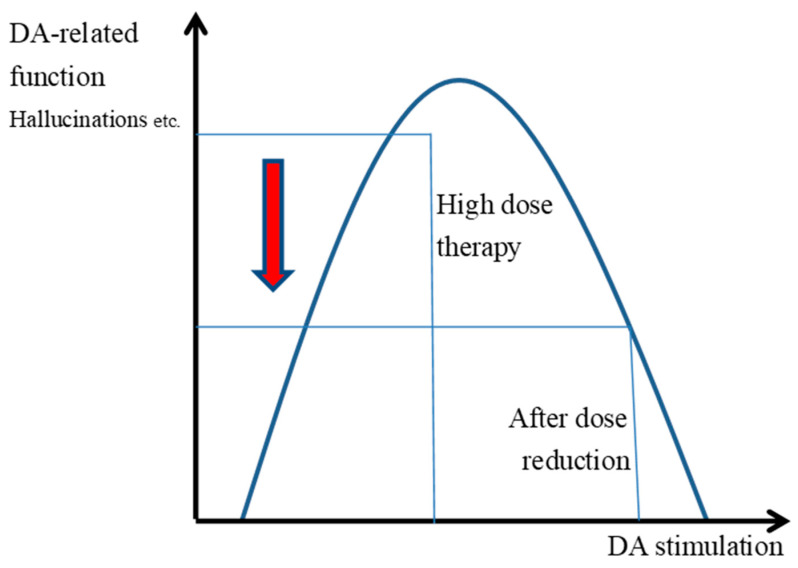
Halving the antipsychotic dose at relapse reduces DA-related function and positive symptoms in patients with schizophrenia.

Psychological, social, and physiological stress, abnormal glutamate transmission, immunodeficiency, inflammation, and reduced neurotrophin levels, such as brain-derived neurotrophic factor, contribute to the release of DA, resulting in elevated extracellular DA levels in striatal medium spiny neurons (MSNs) [21,22]. Increased striatal DA synthesis and elevated extracellular DA concentrations lead to heightened impaired reward-based learning and impulsive behavior, ultimately inducing or exacerbating psychotic symptoms (Figure 1) [12]. Halving the dose of antipsychotics could significantly decrease the DA block or excessive DA stimulation, which would reduce DA function and the positive symptoms (Figure 2).

When the dose of antipsychotics is halved, the DA block is largely reduced and DA stimulation becomes excessive, which may result in a reduction in DA function (Figure 2: the red arrow in the diagram).

Based on this hypothesis, we conducted a clinical trial to assess whether symptoms improved following halving the antipsychotic doses during relapse in patients with schizophrenia receiving high-dose antipsychotic therapy.

## 2. Results

### 2.1. Comparison of the Halved-Dose Group and the High-Dose Group

**Figure 3:**
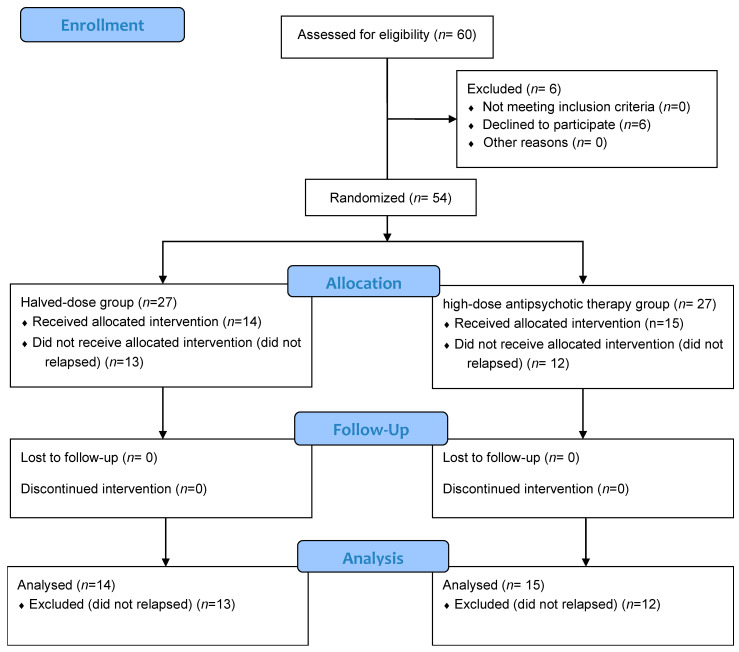
CONSORT flow diagram.

A total of sixty patients were initially included in the study; however, six patients withdrew their participation and were consequently excluded. During the randomization procedure, 54 patients were randomly assigned to the halved-dose group (*n* = 27) or the high-dose group (*n* = 27). The CONSORT flow diagram (Figure 3) provides the full details of the enrollment, allocation, and follow-up.

Based on conventional criteria for treatment-resistant schizophrenia (TRS) [23], 44 of the 60 participants were classified as having TRS. Sixteen patients did not have a PANSS (Positive and Negative Syndrome Scale) score of moderate severity or higher (≥60 points, two or more moderate positive symptoms or one or more severe positive symptoms, and two or more moderate negative symptoms or one or more severe negative symptoms).

**Figure 4:**
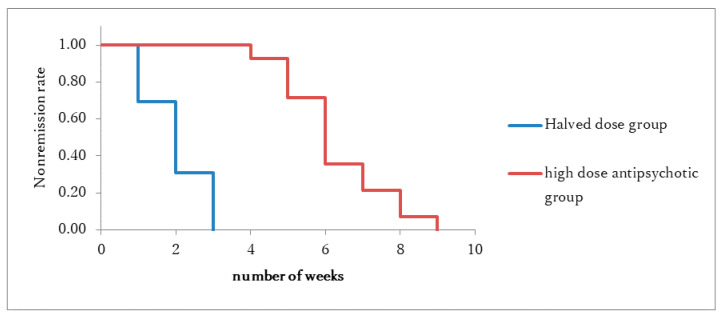
The non-improvement rate in the halved-dose group and high-dose group after relapse.

Among the 54 patients with schizophrenia undergoing high-dose antipsychotic therapy, 29 patients (54%) relapsed, of whom 14 (52%) were in the halved-dose group while 15 (56%) were in the high-dose group. No statistically difference was observed between the two groups (*p* = 0.89, t = 0.14). Notably, all the patients who relapsed subsequently achieved an improvement. In the halved-dose group, the antipsychotic dose was rapidly reduced by half, leading to an improvement within a span of 3 weeks for all patients. Conversely, in the high-dose group, a period of 4–9 weeks was needed for improvement, with a significant difference observed in the period until improvement (*p* <0.001, χ^2^ = 17.24 (log-rank test)) (Figure 4).

The median period until improvement from relapse was 14 days in the halved-dose group (95% confidence interval [CI]: 11, 17) and 42 days in the high-dose group (95% CI: 39, 49). At baseline, no differences were observed between the halved- and high-dose groups in terms of age, male-to-female ratio, antipsychotic dose, PANSS, and DIEPSS (Drug-induced Extrapyramidal Symptoms Scale) (Table 1). The anticholinergic drugs used included biperiden 1–3 mg or trihexyphenidyl 2–6 mg.

In the halved-dose group, there was a significant improvement in the DIEPSS scores after the antipsychotic drug dosage was halved (baseline: 10.8 ± 3.6, after improvement: 8.0 ± 2.7, *p* < 0.001, *t* = 6.6). No significant difference was observed when compared to the high-dose group (Table 2).

### 2.2. Safety

Adverse events related to the treatment were observed in six patients (43%) in the halved-dose group (including constipation in three patients, headache in two, and dry mouth in two); however, none of these adverse events led to therapy cessation, and all patients achieved improvement. In the high-dose group, adverse events were observed in ten patients (73%), including fever in two patients, headache in five, vomiting in two, upper respiratory tract infection in two, dysuria in one, and constipation in three patients. However, all of these events either improved or alleviated, and no adverse events were observed that precluded continuation of the clinical trial. Furthermore, in the halved-dose group, one patient with consistently elevated white blood cells from baseline exhibited improvement.

### 2.3. Changes in PANSS and DIEPSS Scores During Relapse

During relapse, all patients exhibited an aggravation of the PANSS score and an exacerbation or improvement in the DIEPSS score. Hallucinations or delusions were observed in twenty-four patients (83%), irritability and aggressiveness in twenty (69%), and impulsive action in five (17%). Regarding the DIEPSS score, an improvement of 3 points or more was noted in six patients (21%), while aggravation was observed in four patients (14%). Aggravation primarily included akathisia, sialorrhea, gait disturbances, and tremors.

## 3. Discussion

### 3.1. Dopamine (DA) Neuron Firing, DA Transients, and DA Function

The use of antipsychotics is effective for the treatment of positive symptoms of schizophrenia [24]. Antipsychotic therapy primarily targets DA D2 receptors and exerts a therapeutic effect against hallucinations and delusions by blocking them [25]. DAD2 receptor activity in the striatum is associated with reward learning, motivated behavior [26], reversal learning, associative learning, and habit formation [27,28]. DA neurons in the ventral tegmental area (VTA) and the substantia nigra pars compacta respond to rewards by bursts of action potentials [29,30], and the response toward unexpected or unanticipated rewards induces a large increase in firing, whereas for expected rewards, there is almost or absolutely no change in firing. However, when expected rewards fail to materialize, DA neuronal firing is reduced below baseline. It seems that DA neurons encode a reward prediction error and report a discrepancy between the expected reward and the reward obtained [31,32]. Accordingly, DA neurons do not respond to the presence of a given unchanged reward. Furthermore, salient non-reward stimuli also induce DA neuron firing [33], and the mesocortical dopamine pathway (i.e., the VTA–prefrontal cortex (PFC) pathway) is primarily activated, even in response to aversive stimuli [34,35]. These are involved in the reliability, certainty, and accuracy of perceptual tasks [36,37] and reward signal transmission [38,39].

In in vivo studies, extracellular DA fluctuations in the striatum in relation to drug administration and various behavioral events depended on the DA release evoked by the burst firing of DA neurons [40], which is called a DA transient. Spontaneous transients are increased in patients with schizophrenia, and it has been pointed out that this increase might reflect the inappropriate chaotic phasic firing of DA neurons [25,41]. The burst firing of DA neurons might have a clear impact on the intermediation of motivation and learning, while bursts triggered by cues modulate actions in progress and might contribute to future actions via altered synaptic plasticity [42].

### 3.2. Model of Delusion Formation in Schizophrenia

The theoretical model of the psychotic symptoms of schizophrenia constitutes the hypothesis that mesolimbic dopamine signals contribute toward the salient marking of environmental stimuli and that excessive DA transients signify internal and external irrelevant stimuli [25,43]. Assuming that DA release evoked by the phasic firing of neurons reflects sensory stimulation, nonrewarding salient stimuli, and the attribution of the incentive salience of internal representation [44], it is conceivable that the abnormal firing of dopaminergic neurons arising from DA dysregulation, which imbues irrelevant stimuli with meaning—called aberrant salience attribution—leads to the development of delusions in schizophrenia [25,45].

In the learning process, DA functioning is involved in encoding information about values, prediction error, and information accuracy where DA neuron firing is expected [46]. Uncertainty affects the prediction error caused by erroneous weighting and wrong reasoning [47,48], and abnormal DA signaling increases uncertainty and has an adverse effect on learning. Abnormal decision making with uncertainty is an important cause of delusion formation in the case of psychosis [49].

Dopaminergic dysfunction in the dorsal striatum associated with the recognition of habit orientation might result from strong thought patterns with abnormal thought content [50], and once combined with dopaminergic activity, the ability to evoke dopaminergic activity is maintained over time, even if the stimulus lacks inherent salience [51]. Once psychotic symptoms are induced, even after a long period of stability, DA perturbation is evoked upon use of a single dose of a psychostimulant, such as amphetamines.

### 3.3. Increased DA Release and Relapse in Patients with Schizophrenia

Patients with schizophrenia present increased presynaptic striatal DA synthesis (7), and during relapse, there is an increase in DA release from DA neurons as well as in extracellular DA concentrations [9,10,11,52]. Increased striatal DA synthesis and extracellular DA concentrations induce and exacerbate psychotic symptoms [12]. Increased DA release in the striatum correlates with auditory hallucinations [53,54], and increased DA release caused by DA agonists correlates with the exacerbation of positive symptom severity [15,50] and exacerbated positive symptoms [55].

The striatum is the center of the cortical–striatal–thalamic–cortical circuit and serves as an interface for sensory inputs and their cortical targets [43,56]. D1 and D2 receptors expressing MSNs in the associative striatum project to the thalamus directly and indirectly and complete a feedback and feedforward loop with the cortex [56]. In the presence of sensory signals, as a clue to distinguish the results of one’s motor act and environmental changes, a motor signal copy (efference copy) is generated in the cortex. The glutamatergic neurons that transmit these signals form synapses in DAD2-expressing MSNs in the striatum [57,58]. Increased DA release in the striatum results in excessive dopaminergic signaling, which impedes the appropriate transmission of efferent copies and, therefore, makes it difficult to distinguish self-evoked sensory changes from external changes. It has been previously pointed out that these sensory aberrations contribute toward the development of the passivity phenomena and auditory hallucinations [57]. Furthermore, DA release controls striatal MSN activity [59]; therefore, it is inferred that excessive DA signaling in the striatum directly leads to the onset of inappropriate behaviors and psychotic symptoms. Dopaminergic signaling in the striatum controls competing behavior by activating the MSNs of the D1 direct pathway and suppressing the activity of the MSNs of the D2 indirect pathway [60]. In the striatum, when the DA release is increased, it becomes difficult to control competing behavior, making it difficult to carry out appropriate behavior and easier to carry out inappropriate behavior [61]. According to a study of patients with Parkinson’s disease, DA agonists caused sensory and behavioral changes and increased the risk of developing impulse control disorder [54].

Antipsychotics reduce excessive D2 signaling and control D1 receptor MSN and D2 receptor MSN activities [62], which might reduce psychotic symptoms and inappropriate behaviors. It is possible that increased DA release in the striatal synapses might lead to response changes in the striatum (transients) or specific changes in the basal ganglia and cortical function, thereby causing relapse.

### 3.4. The Inverted U-Shaped Relationship Between the Transmission and Functioning of DAD2 Receptors

The level and function of neurotransmitters in DAD1 receptors of the frontal lobe, as well as in α-1 and β-1 epinephrine receptors, present an inverse U-shaped relationship, and if there is too much or too little DA and epinephrine, the transmitter-mediated function will not be optimal. Stimulation of the α-1 receptor by high levels of NE reduces PFC cell firing, resulting in impaired PFC function [63]. Similarly, excess or insufficient stimulation of the α-2A receptor reduces PFC cell firing [64,65]. Reduced PFC function impairs cognitive functions such as working memory, flexible thinking, and attention [64]. The clinical antipsychotic agents used for schizophrenia treatment have different affinities for the α-1 and α-2 receptors. It would be difficult to simultaneously optimize the stimulation of the α-1 and -2 receptors when prescribing high-dose and combination antipsychotics, and high levels of NE could cause some impairment of cognitive functions.

The level and performance of acetylcholine (Ach) receptors in the cortex also show an inverted U-shaped reaction response [66]. In healthy adults, upon administering bromocriptine, which is a DAD2 receptor agonist, it was found that the performance of subjects with high DA synthesis in the striatum in a reversal learning task was reduced, as a result of overstimulation by bromocriptine [6]. Most patients with early-stage Parkinson’s disease sustain damage to the putamen of the dorsal striatum; however, the ventral striatum remains relatively undamaged. Therefore, the dose of levodopa that has a beneficial effect on motor tasks such as instrumental learning, which mostly depends on the putamen on the dorsal side [7], and cognitive tasks, such as set-shifting [67], causes excessive stimulation in the ventral striatum and reduces the performance in cognitive and motor tasks, such as reversal learning tasks, that depend on the ventral striatum [40]. In an animal experiment, an inverted U-shaped dose–response relationship was found between changes in the activity of the dorsolateral striatum and behavior of rats according to the dose of amphetamines [68]. In a different experiment, it was found that receptor stimulation by quinpirole, which is a DA agonist of DAD2 receptors, typically caused robust long-term depression (LTD) in long-term potentiation (LTP) induction protocols [69]. Adaptive striatal DA transients are stimulated toward reward-predicting cues under the effect of moderate doses of amphetamine, whereas, at high doses, these transients become dull [70].

### 3.5. Symptom Changes and Improvement Associated with Halving the Dose of Antipsychotics

The striatum is involved in perceptual and ideational symptoms of schizophrenia [71], and there is abundant evidence indicating that, as described above, excess DA in the striatum correlates with the activation of delusions and auditory hallucinations. Reduced striatal dopaminergic activity in patients with schizophrenia using antipsychotics is consistent with reduced goal-oriented behavior and the slowing of psychomotor functions [20,72].

High-dose antipsychotic therapy blocks DAD2 receptors to a high degree, and in patients with schizophrenia at times of remission (Figure 1), the appearance of hallucinations and delusions is less frequent, and inappropriate motivated behavior is also reduced. When relapse occurs, the release of DA as well as the extracellular levels of DA in the striatal MSN increase. The predominant binding of antipsychotics to DAD2 receptors during remission is reduced by increased DA concentrations during relapse, and the D2 receptor transmission might increase with the increased binding of the DA and DAD2 receptors (Figure 1). It is inferred that increased DA transmission causes an increase in erroneous actions, false motivation, and impulsive actions.

### 3.6. Increased Stress Exacerbates Relapse

High-dose antipsychotic therapy is associated with various adverse effects due to anti-DA, anticholinergic, antihistaminic, and anti-adrenergic effects. Elevated antipsychotic D2 receptor occupancy contributes to dysphoria, depression, and the exacerbation of negative symptoms [73,74]. Additionally, the blockade of more than 78% of the D2 receptor increases the risk of extrapyramidal symptoms (EPS) [75]. Increased anticholinergic activity causes gastrointestinal disturbance and urinary retention, while increased antihistaminic and anti-adrenaline actions result in sedation and reduced concentration. These dysphoria and disturbances in physical and cognitive functioning serve as negative or aversive stimuli, i.e., stress, for patients. Whether acute or chronic, they are perceived as stress in the hypothalamus, promoting norepinephrine (NE) release from the locus coeruleus and cortisol release from the adrenal cortex. Increased cortisol evokes DA release [76].

### 3.7. Increased Stress Impairs Prefrontal Cortex Functioning

While striatal activity increases during relapse, the PFC function is reduced, resulting in decreased cognitive function and a propensity for inappropriate actions. Reduced PFC function due to stress causes the recurrence of various inappropriate actions, including a loss of self-control, drug dependence, smoking, alcohol consumption, and excessive eating [77]. The ventral medial PFC regulates emotional responses through its extensive connections with the nucleus accumbens (ventral striatum) and amygdala, subcortical structures responsible for emotional responses and habits [78].

The release of the catecholamines NE and DA in the PFC is related to an arousal state, attention, behavior, and emotion. α-adrenergic receptors in the PFC exhibit an inverted U-shaped performance curve. In states of stress, rapidly released high levels of NE decrease PFC neuron firing and reduce NE-related functions through the α1-adrenergic receptor stimulation [79]. Similarly, the relationship between the stimulation of DAD1 receptors in the PFC and working memory and cognitive control exhibits an inverted U-shaped curve; under stress conditions, the overstimulation of D1 receptors inhibits the firing of delay cells and consequently reduces the PFC function [80]. Excessive catecholamine weakens PFC function and enhances activity in the primary visual cortex, striatum, and amygdala; the orchestration of the brain responses switches from deliberative PFC regulation to habitual responses in the basal ganglia and reflexive responses in the amygdala [81].

Reducing the antipsychotic dose contributes to an improvement in physical functioning, such as walking difficulty, tremor, and gastrointestinal disturbances, and subjective well-being, including malaise and dysphoria, coupled with enhanced cognitive function. The elimination of these adverse effects leads to a reduction in cortisol and catecholamine release, thereby restoring the PFC function and potentially inhibiting relapse. Studies have reported a correlation between cortisol suppression and mesolimbic DA transmission, suggesting that cortisol suppression may have effects similar to those of antipsychotics [82].

### 3.8. Improved Functioning of DAD2 Autoreceptors Acts to Improve After Relapse

D2 autoreceptors, expressed in DA neurons, exhibit negative feedback regulation by reducing DA neuron firing, synthesis, and release. Stimulation of D2 autoreceptors results in a reduction in the induced DA release, a process that is partially blocked by antipsychotics. In DA neuron autoreceptor knockout mice, the absence of these autoreceptors led to feedback inhibition, resulting in increased DA synthesis and release [83]. The blockade of D2 autoreceptors by high-dose antipsychotics inhibits feedback, making it challenging to regulate DA release. However, halving the dose of antipsychotics may reactivate the feedback mechanism, suppressing excessive DA synthesis and reducing DA release.

Comparing this trial and the long-term reductions in high-dose therapy, the effect size (ES) for improvement in symptoms and functions was 0.1 for both approaches, but the improvement in EPS was 0.8 for this trial and 0.3 for the long-term reduction [84,85,86]. No serious adverse events and a 10% dropout rate were reported with this trial dose reduction, while the dropout rate with long-term dose reductions ranged from 7 to 31% [84,85,86]. Our approach to dose reduction applied only to patients who relapsed, while long-term dose reduction was reserved for those in remission. Halving the dose of antipsychotics has shown to have therapeutic effects against relapse and reduce adverse events. Furthermore, reducing the dose of antipsychotics offers the advantage of making an increase in antipsychotic dose a viable treatment option in the event of a future relapse, which can be considered a positive aspect of this treatment method.

The approach of treating relapse by halving the dose of antipsychotics is not exclusive to high-dose antipsychotic therapy. It may potentially be effective for addressing relapse in patients using antipsychotics at a relatively high CP-equivalent dose of 1000 mg and those believed to have elevated DAD2 receptor occupancy due to antipsychotics. Further studies are needed to confirm this hypothesis.

## 4. Methods

### 4.1. Study Design

This clinical trial constituted a two-group, randomized, and single-blind study aimed at evaluating relapse outcomes in patients with schizophrenia undergoing high-dose antipsychotic therapy. Patients were blinded. Monitors were not blinded. The study compared the time to improvement between the halved-dose and high-dose antipsychotic therapy groups.

### 4.2. Ethics Approval and Recruitment

The protocol received approval from the ethical review board of Showa University (approval number: 20H073), and signed informed consent was obtained from all study participants after a verbal and written explanation of the protocol. The participants, who were inpatients at Edogawa Hospital between 2021 and 2022, were enrolled in the study, performed in accordance with the Declaration of Helsinki.

### 4.3. Participants

The participants comprised individuals aged 20–70 years with schizophrenia according to the Diagnostic and Statistical Manual of Mental Disorders, Fifth Edition (DSM-5). The inclusion criteria encompassed participants who were informed about the clinical trial, open to the prospect of reducing antipsychotic medication, and presently in a state of remission while undergoing high-dose antipsychotic therapy. The term high dose was defined as oral therapy with a dose equivalent to 1000 mg or more of CP.

The exclusion criteria comprised individuals aged < 20 years and ≥70 years and those with concurrent neurodevelopmental and organic disorders, including symptomatic and mental disorders. Additionally, individuals who had undergone long-term depot antipsychotic treatment within the preceding 3 months, those who received electroconvulsive therapy (ECT) within the past 6 months, and those with a history of substance dependence were excluded.

### 4.4. Sample Size

Regarding the sample size calculation for the log-rank test, with a significance level of α = 0.05 and a power of 0.8, the sample size for one group was determined to be 14.02 using the Freedman formula and 11.61 using the Schoenfeld formula. Assuming a relapse rate of 0.5 for patients with schizophrenia undergoing high-dose antipsychotic therapy over 1 year, the required recruitment numbers were calculated as 56 and 46, respectively. To account for the potential withdrawal of informed consent, the final sample size was set to 60.

### 4.5. Randomization

Beforehand, an independent personal information manager categorized all patients in the halved-dose and high-dose groups into blocks of four cases, maintaining a 1:1 ratio (two cases vs. two cases). Utilizing an allocation code table, “assignment instructions and confirmation forms” were generated and sealed in envelopes. Subsequent to obtaining informed consent, the attending physician performing the study, unsealed the envelope, and verified the allocation for research intervention based on the “assignment instructions and confirmation form”.

### 4.6. Primary Outcome

The primary outcome measured in the study was the duration from relapse to improvement in the halved-dose and high-dose antipsychotic therapy groups.

### 4.7. Secondary Outcomes

The study’s secondary outcomes included post-improvement psychiatric symptoms, EPS attributed to antipsychotics, and adverse events resulting from halved-dose or high-dose antipsychotic therapy. Psychiatric symptoms were assessed using the Positive and Negative Syndrome Scale (PANSS), while EPS was measured using the Drug-induced Extrapyramidal Symptoms Scale (DIEPSS).

### 4.8. Intervention

#### 4.8.1. Antipsychotic Dose Reduction

The antipsychotic dose was generally reduced in a single adjustment. Antipsychotics administered at a CP-equivalent dose (mg CP/day) ranging from 1000 to 2000 mg CP/day were reduced to approximately 700–1000 mg CP/day. For antipsychotics prescribed at a dose of 2000 mg CP/day or more, the reduction target was approximately 1000–1500 mg CP/day. High-potency antipsychotics, such as risperidone, paliperidone, and haloperidol, were the specific drugs targeted for dose reduction.

Patients for whom dose reduction was slow (2–3 times/2–3 weeks) included the following categories: (1) patients with minimal EPS, characterized by a baseline total DIEPSS score < 5 points, or those without one or more items with moderate (3 points) or higher scores; (2) patients predominantly using antipsychotics with potent anticholinergic effects (quetiapine, olanzapine, CP, levomepromazine, zotepine, or blonanserin); (3) patients exhibiting fewer than two anticholinergic adverse effects, such as tachycardia, constipation, nausea, vomiting, decreased appetite, ileus, thirst, urinary retention, and dizziness.

In cases where any of the conditions stated above applied, and if psychotic symptoms were serious, posing imminent danger, priority was given to rapid dose reduction for symptom improvement. The antipsychotic dose was reduced by a single dose reduction of approximately 30%. Even if the antipsychotic dose did not reach the targeted range of 1000–1500 mg CP/day after a single reduction, the dose reduction was considered complete if there improvement from relapse was observed.

#### 4.8.2. Anticholinergic Dose Reduction

The dose of anticholinergics was reduced at the discretion of the attending physician subsequent to the reduction in the antipsychotic dose.

#### 4.8.3. Relapse

Relapse was defined in instances where one or more of the following criteria were met: (1) an increase of 25% or more in the total PANSS score, (2) deliberate self-injury, (3) suicidal or homicidal ideation that was deemed clinically significant, and (4) violent behavior resulting in clinically significant injury to another person or property.

#### 4.8.4. Remission

Remission was defined as a PANSS score of 3 or less on 8 items, including delusions, unusual thought content, and hallucinatory behavior, lasting at least 6 months [87].

### 4.9. Data Analysis

Survival analysis was performed using the Kaplan–Meier method, assessing the period from relapse until improvement, defined as the non-improvement rate. Comparison was conducted using log-rank analysis. The demographic data, PANSS, DIEPSS, and antipsychotic doses between the halved-dose and high-dose antipsychotic therapy groups were compared using a *t*-test before and after relapse. Data analysis was performed using the BellCurve for Excel (by Social Survey Research Information Co., Ltd., Tokyo, Japan).

### 4.10. Safety Monitoring

Blood pressure, temperature, pulse, and respiration rate were measured daily, psychiatric symptoms and side effects were assessed at least weekly, and the PANSS and DIEPSS were administered at baseline, when symptoms worsened, and when symptoms improved. Regarding compliance with oral medications, the nurse made sure that they were taken internally.

## 5. Conclusions

Halving the antipsychotic dose was associated with a shorter period from relapse to improvement. Currently, there is a lack of information regarding the efficacy and tolerability of treatment for relapse in patients with schizophrenia receiving high-dose antipsychotic therapy. The sample size and statistical power of this study were insufficient to provide information that would contribute to more rational treatment decisions. Therefore, after addressing methodological issues, further studies with better reproducibility and larger sample sizes are required to prove the generalizability of the results of this study.

This was a clinical trial conducted under the unusual circumstances of a large number of long-term hospitalized patients and high-dose antipsychotic therapy patients in Japan. Rater training was provided, but interrater reliability coefficients were unknown.

### Limitations

When interpreting these study results, attention should be paid to limitations such as the small sample size and limited statistical power.

## Figures and Tables

**Table 1:** Demographic variables and clinical status of patients with schizophrenia at baseline (M ± SD) (halved-dose group (*n* = 27) and high-dose group (*n* = 27)).

	Halved-Dose Group (*n* = 27) (Number of Relapse = 14)	High-Dose Group (*n* = 27) (Number of Relapse = 15)	T (Relapsed Patients)	*p*-Value (Relapsed Patients)
Age	50.2 ± 10.5 (49.6 ± 7.9)	47.6 ± 10.0 (45.2 ± 9.3)	0.90 (1.27)	0.37 (0.22)
Sex	Male: 14 (6) Female: 13 (8)	Male: 14 (7), Female: 13 (8)	0.00 (0.37)	1.00 (0.71)
Dose of antipsychotics (CP eq./mg)	1695 ± 492 (1793 ± 393)	1618 ± 429 (1836 ± 413)	0.54 (0.26)	0.59 (0.79)
PANSS	66.5 ± 13.1 (68.7 ± 12.6)	68.3 ± 11.2 (70.4 ± 9.8)	0.37 (0.86)	0.71 (0.40)
DIEPSS	10.2 ± 5.8 (10.8 ± 3.6)	9.5 ± 4.8 (9.3 ± 4.8)	0.87 (0.76)	0.39 (0.46)

The values indicate mean ± SD or number of participants. *t*-test: α = 0.05; CP eq.: chlorpromazine equivalent.

**Table 2:** PANSS, DIEPSS, and antipsychotic drug dose at improvement following relapse (after treatment for relapse).

	Halved-Dose Group	High-Dose Group	T (Relapsed Patients)	*p*-Value
PANSS	69.2 ± 6.8	68.1 ± 7.2	0.11	0.91
DIEPSS	8.0 ± 2.7	9.9 ± 4.2	0.88	0.12
Dose of antipsychotics (CP eq./mg)	1014 ± 241	1771 ± 454	4.83	<0.001

Values are mean ± SD; *t*-test: α = 0.05; CP eq.: chlorpromazine equivalent.

## Data Availability

The datasets used and/or analyzed during the current study are available from the corresponding author upon reasonable request.

## List of Abbreviations

DA	Dopamine
PFC	Prefrontal cortex
CP	Chlorpromazine
PANSS	Positive and Negative Syndrome Scale
EPS	Extrapyramidal symptoms
DIEPSS	Drug-induced Extrapyramidal Symptoms Scale
NE	Norepinephrine
